# The Impact of Developmental and Metabolic Cues on Cytoophidium Formation

**DOI:** 10.3390/ijms251810058

**Published:** 2024-09-19

**Authors:** Yuanbing Zhang, Ji-Long Liu

**Affiliations:** 1 School of Life Science and Technology, ShanghaiTech University, Shanghai 201210, China; 2 Center for Experimental Medicine, The First Affiliated Hospital, Jiangxi Medical College, Nanchang University, Nanchang 330006, China; 3 Shanghai Clinical Research and Trial Center, Shanghai 201210, China; 4 Department of Physiology, Anatomy and Genetics, University of Oxford, Oxford OX1 3PT, UK

**Keywords:** cytoophidium, CTP synthase, compartmentation, metabolic regulation, developmental cue

## Abstract

The cytoophidium, composed mainly of CTP synthase (CTPS), is a newly discovered dynamic filamentous structure in various organisms such as archaea, bacteria, and humans. These filamentous structures represent a fascinating example of intracellular compartmentation and dynamic regulation of metabolic enzymes. Currently, cytoophidia have been proven to be tightly regulated and highly dynamic, responding rapidly to developmental and metabolic cues and playing a critical role in maintaining cellular homeostasis. In this review, we would like to discuss in detail the characteristics, mechanisms, functions, and potential applications of this conservative but promising organelle.

## 1. Introduction

In 2010, we independently discovered, with two other groups, that cytidine 5′-triphosphate (CTP) synthase (CTPS), an essential metabolic enzyme responsible for the CTP de novo synthesis, can form elongated, membrane-less filamentous structures in fruit fly [1], bacterium [2], and budding yeast [3]. Therefore, we named it cytoophidium (Greek for “cellular snake”, plural cytoophidia). Subsequently, CTPS cytoophidia were observed in mammalian cell [4,5], fission yeast [6], plant [7], zebrafish [8] and archaea [9]. The discovery of cytoophidia in archaea, bacteria, and eukaryotes indicates that they are extremely conserved in evolution.

The synthesis of CTP encompasses both de novo and salvage synthesis pathways. CTPS is the rate-limiting enzyme responsible for the ATP-dependent transfer of cytoplasmic amido-nitrogen from glutamine to the C-4 position of the UTP to generate CTP, ADP and glutamate [10]. Its product CTP is essential for cellular metabolism and functions in membrane phospholipid synthesis [11,12,13], the building block for RNA [14], and synthesis of the glycoprotein precursor dolichol phosphate [15]. CTPS can directly bind to all four ribonucleotides: ATP, CTP, UTP, and GTP [16]. These four nucleotide pools also control CTPS activity, highlighting the significance of CTPS in nucleotide metabolism.

Notably, enzymatic filamentation is a fascinating aspect of cell biology, and this process has also been observed in other metabolic enzymes. The formation of cytoophidia emerges as a novel mode of enzyme regulation in cellular metabolism that facilitates cells to adapt to changes in nutrient availability and other metabolic cues.

In this review, we will introduce the characteristics and structure of the cytoophidium, which is a conserved filamentous structure in cells. Additionally, we will discuss the dynamic assembly, functions, and potential applications of cytoophidium formation. Finally, we will present our perspectives for further investigation into cytoophidia.

## 2. Characteristics of Cytoophidia

**Figure 1:**
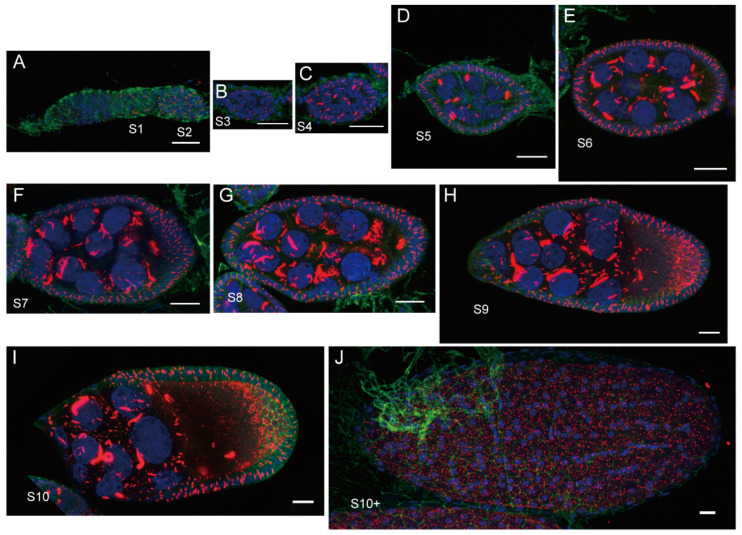
Schematic representation of cytoophidia formation at the stages of *Drosophila* oogenesis. (**A**–**J**) Red represents mCherry-tagged CTPS, green for cell membranes, and blue for nuclei. S: stages. Scale bars: 20 μm.

The cytoophidium has the following characteristics: a filamentous structure in morphology, primarily composed of metabolic enzymes, and a membrane-less cellular structure [17]. Cytoophidia have been discovered in *Drosophila*, with many results indicating that the assembly of cytoophidia in *Drosophila* is tightly regulated and highly dynamic (Figure 1).

Cytoophidia appear as membrane-less serpentine filaments within the cytoplasm. CTPS monomers contain an α-helical linker connecting an amidoligase domain at the N-terminal end to a glutamine amidotransferase domain at the C-terminal end [18,19]. Dimerization of CTPS dimers forms tetramers, which are then polymerized into metabolic filaments and higher-order bundled assemblies. Several structural analyses have revealed that CTPS can bind to ribonucleotides (NTPs) to form filaments in bacteria [20,21], yeast [22], fruit flies [23], and humans [24,25,26]. Recently, we discovered that *Drosophila* CTPS can also form filaments with dNTPs [27]. In a separate review, we summarize the current structural understanding of CTPS and cytoophidia [28].

The polymerization of cytoophidia is speculated to be delineated into five stages: nucleation, elongation, fusion, bundling, and circularization [17]. The abundance and morphology of cytoophidia exhibit variation in distinct cells in different periods. Dot, linear, circular, and C-shaped forms are observed depending on cellular states. Cytoophidia can be classified into macro-cytoophidia and micro-cytoophidia based on their relative sizes [1]. Additionally, cytoophidia has been observed both in the cytoplasm and nucleus, and micro-cytoophidia can undergo multiple rounds of fusion to form macro-cytoophidia [29].

Inosine monophosphate dehydrogenase (IMPDH), the rate-limiting enzyme in the GTP de novo synthesis pathway, can also form filamentous structures. Super-resolution imaging showed that CTPS cytoophidia coordinate and intertwine with IMPDH cytoophidia in a mixed filamentous structure [30]. Asparagine synthetase (ASNS) can form cytoophidia in yeast [31]. ∆1-pyrroline-5-carboxylate synthase (P5CS), a key enzyme involved in proline (Proline) synthesis, forms P5CS cytoophidia in *Drosophila* [32,33] and *Arabidopsis* [34]. Acetyl-CoA carboxylase, a rate-limiting enzyme in fatty acid biosynthesis, exists as polymeric filaments in two distinct activated and inhibited forms [35,36,37,38]. In addition, a genome-wide screen in yeast identified 23 proteins that can form filamentous structures, mainly metabolic enzymes [39], suggesting that the formation of cytoophidia by metabolic enzymes is a novel yet widespread form of intracellular compartmentalization (Table 1).

## 3. Mechanism of Cytoophidium Formation

**Figure 2:**
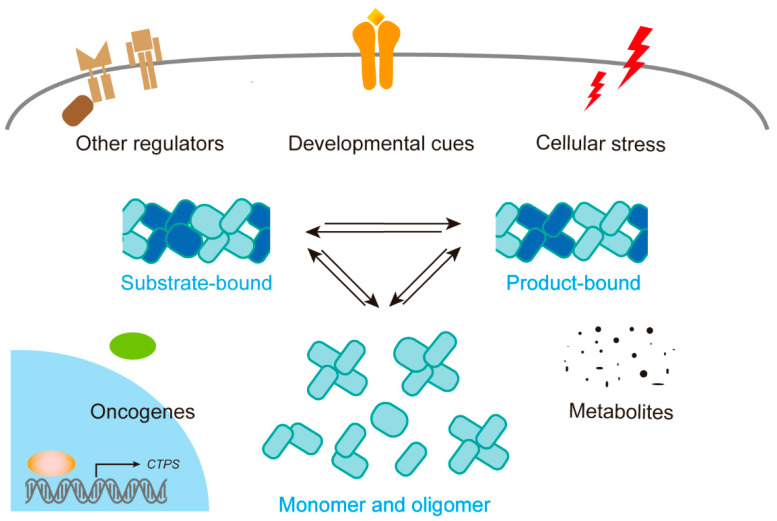
Regulation of cytoophidium formation. The polymerization of CTPS into cytoophidia is affected by several factors, including cellular metabolites, cellular stress, developmental cues, proto-oncogenes, and other regulators.

The polymerization of CTPS into cytoophidia is influenced by various factors such as cellular metabolites, cellular stress, developmental cues, proto-oncogenes, and other regulators (Figure 2 and Table 2). Therefore, their formation is reversible, indicating a dynamic response to changes in the cellular environment.

### 3.1. Cellular Metabolites

Glutamine analogs such as 6-diazo-5-oxo-l-norleucine (DON) and azaserine promote cytoophidium formation in various *Drosophila* tissues and human cells [4]. Subsequently, several studies also found that glutamine deprivation and treatment with its analogs promote the formation of cytoophidia [29,62,63]. Further, the CTP analog gemcitabine-5′-triphosphate (F-dCTP), a potent inhibitor of CTPS, has also been used as a chemotherapeutic metabolite, which induces the formation of cytoophidia [64]. Yeast undergoes cytoplasmic acidification upon starvation, and this pH-sensitive assembly mechanism is also found in budding yeast CTPS [22].

### 3.2. Cellular Stress

Cytoophidia form in *S. pombe* during the exponential phase and break down during the stationary phase. In contrast, *S. cerevisiae* displays cytoophidia during the stationary phase, which disappear during the exponential phase [60,65]. Further, cold or heat shock rapidly and reversibly decrease the length and frequency of cytoophidia in fission yeast but not in budding yeast [66]. Carbon depletion induces cytoophidium formation in budding yeast while leading cytoophidia dispersion in fission yeast [3]. Additionally, hypoosmolality impedes cytoophidium integrity during nitrogen starvation in fission yeast, indicating that the culture environment affects cytoophidia [67].

In *Drosophila* ovaries, cytoophidia elongate in response to nutrient deprivation and heat shock [68]. Nutritional stress also induces cytoophidium formation in other *Drosophila* tissues, and its formation is reversible upon refeeding [51]. Inhibition of glycolysis disrupts the cytoophidia structure and impairs cell proliferation [69]. Mechanically, starvation stress and glutamine deficiency activate the GCN2/ATF4/MTHFD2 axis, ultimately coordinating the formation of cytoophidia [52].

### 3.3. Developmental Cues

During development, the formation and function of cytoophidia can provide insights into cellular metabolism and its regulation. The length of cytoophidia in *Drosophila* follicle cells in egg chambers varies significantly at different stages, correlated with Myc protein levels [53]. During *Drosophila* oogenesis, the length of cytoophidia in follicle cells gradually increases up to stage 10A, and after stage 10B, they slowly decrease until they disappear [1,70]. CTPS down-regulation suppresses the overgrowth phenotype caused by Myc overexpression, indicating that CTPS functions downstream of Myc and is necessary for Myc-mediated cell size control [53].

Cytoophidia are also prevalent in dormant neuroblasts and break down after reactivation in the *Drosophila* larval central nervous system [51]. Cytoophidia exist in intestinal stem cells (ISCs) and enteroblasts in the *Drosophila* midgut [71,72]. In addition, the disruption of CTPS cytoophidia or knockdown of CTPS both inhibit intestinal stem cell proliferation triggered by dextran sulfate sodium [71].

In mice, CTPS cytoophidia are visualized by a specific thymocyte population ranging between DN3 and early DP stages, which undergo rapid cell proliferation [69]. Therefore, the filamentous structure of cytoophidia formed by CTPS can rapidly alter enzyme activity, effectively responding to the metabolic needs of cells at different developmental stages.

### 3.4. Proto-Oncogenes

CTPS forms the cytoophidium in various human cancers [73], and several proto-oncogenes regulate cytoophidia formation in *Drosophila*. The non-receptor tyrosine kinase DAck, the *Drosophila* homologue of mammalian Ack1 (activated cdc42-associated kinase 1), localizes to CTPS filaments and regulates cytoophidia assembly [56]. Cbl, an E3 ubiquitin ligase, is required for cytoophidia formation [57]. CTPS overexpression could rescue the endocycle defect in Cbl mutant cells [57]. Furthermore, a cohort of deubiquitinating enzymes are required for the integrity of cytoophidia in fission yeast [58]. Further, reducing Myc levels leads to cytoophidium loss and smaller nuclear size in follicle cells, whereas Myc overexpression has the opposite effect [53,74]. Recently, it was discovered that Ras^V12^ overexpression increases both the length and abundance of cytoophidia in *Drosophila* ISC. Moreover, the down-regulation of CTPS in Ras^V12^-overexpressing flies decreases the number of proliferating cells [54].

### 3.5. Other Regulators

Several developmental signal pathways have been implicated in the regulation of cytoophidia formation. Inhibition of the mTOR pathway attenuates cytoophidium formation in mammalian and *Drosophila* cells [49]. In contrast to mammalian systems, both TORC1 and TORC2 sub-complexes participate in regulating cytoophidia formation [50]. Additionally, the knockdown of AKT1, which subsequently activates TOR, increases the number of cytoophidia in *Drosophila* neuroblasts [51].

Cytoophidium formation changes periodically with the cell cycle, and histone chaperone Slm9 [59] and histidine-mediated methylation [52] are required for cytoophidia assembly. Further, cytoophidium-high tumors show significantly higher HSP90 expression levels than cytoophidium-negative tumors, suggesting that these cytoophidium-expressing cells might be more aggressive or tolerant to cell stress [73].

The active transport of cytoophidia in fission yeast is regulated by Myo52 (Myosin V), which exhibits a non-tubulin regulatory pattern [60]. Cytoophidia exhibit a polarized distribution on the basolateral side, which is primarily regulated by apical polarity regulators in *Drosophila* epithelial follicle cells [61].

## 4. Biological Functions of Cytoophidia

**Figure 3:**
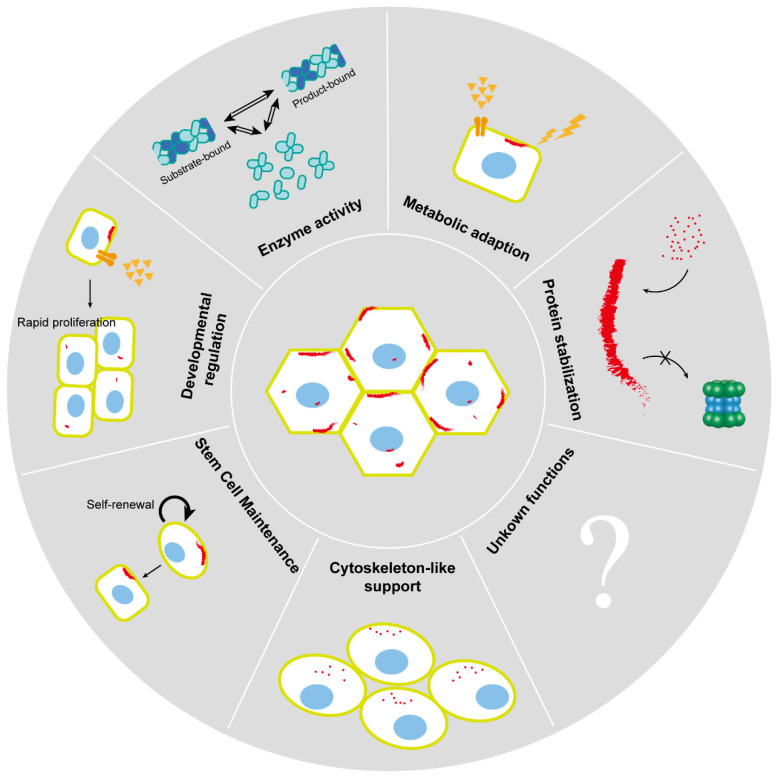
Cytoophidium functions. Cytoophidia are essential for various cellular processes, such as regulating enzyme activity, facilitating metabolic adaptation, supporting developmental regulation, maintaining stem cell function, providing cytoskeleton-like support, and stabilizing proteins.

Characterizing cytoophidia involves a multifaceted approach, integrating microscopy, genetic manipulation, biochemical assays, and live-cell imaging. Notably, cytoophidia play crucial roles in various cellular processes, including regulating enzyme activity, facilitating metabolic adaptation, contributing to developmental regulation, maintaining stem cell function, providing cytoskeleton-like support, and stabilizing proteins (Figure 3).

### 4.1. Enzyme Activity

Enzymatic polymerization refers to a novel mechanism of enzyme kinetics regulation, which may integrate metabolic signaling pathways and play its unique role in nutritional stress, growth, and development. Four NTPs directly bind and control CTPS activity [16]. In bacteria, the inactive *E. coli* CTPS dimers oligomerize into an active tetrameric form with the supplement of substrates UTP and ATP [75]. ATP facilitates the UTP-dependent tetramerization of CTPS, while its product CTP inhibits enzyme activity and prompts shifting into an inactive tetrameric state in *S. cerevisiae* [76]. GTP stimulates the reaction at low concentrations but inhibits it at higher levels [77].

Interestingly, the regulation of CTPS enzyme activity through its polymerization into cytoophidia varies in different species. In bacteria, the cytoophidium is composed of product CTP-bound CTPS and is in an inactive form [21]. Additionally, highly ordered filament bundles that stabilize an inactive state are also found in budding yeast CTPS [22]. In contrast, the human CTPS1 cytoophidia are composed of substrate-bound active CTPS1 and can significantly increase enzyme activity, while the addition of CTP leads to their depolymerization [26]. However, the *Drosophila* CTPS cytoophidia and the human CTPS2 cytoophidia can form both product-bound cytoophidia and substrate-bound cytoophidia in both conformations [23,25]. Metabolic enzymes can either inhibit their active sites or accelerate enzyme activity through filamentous structures.

### 4.2. Metabolic Adaption

Cytoophidium structures can respond to cellular metabolic states, which is particularly important during periods of rapid cell division and differentiation in development. CTPS can mediate cell adhesion in adipose tissue through integrin-dependent mechanisms via its cytoophidia structure. The dynamic assembly of cytoophidia can effectively regulate the PI3K-fatty acid synthase pathway [78].

The depolymerization of cytoophidia can prevent lipid accumulation in adipocytes induced by a high-fat diet through the PI3K-FASN signaling pathway, which indicates that cytoophidia can function as sensors of cellular nutritional status [78]. Understanding the relationship between cytoophidia and metabolic sensing provides insights into how cells adapt to changes in nutrient availability and other metabolic cues.

### 4.3. Developmental Regulation

CTPS and cytoophidia are associated with organ growth and development. In zebrafish, inhibition of CTPS during early development leads to spinal curvature and fluid retention in multiple tissues [79]. In the neuroepithelial stem cells of the *Drosophila* optic lobe, a large number of cytoophidia are present, and overexpression of CTPS impairs optic lobe development [80]. Additionally, in mice, overexpression of CTPS induces the formation of cytoophidia and impairs neuronal migration. Also, the increase in cytoophidia accelerates neuronal differentiation and inhibits the proliferation of neural progenitor cells [81].

In *Drosophila* ovaries, expression of the H355A point mutation, which deprives cells of the ability to form cytoophidia, leads to reduced egg production in females [82]. Moreover, cytoophidia are often found in highly proliferative cells [72], suggesting their potential as cancer targets. Upregulation of CTPS protein levels enhances cytoophidia assembly, and in *Drosophila* testes, overexpression of CTPS induces abnormalities in several oncogenes, causing an enlarged testis head [83].

### 4.4. Stem Cell Maintenance

In certain model organisms, such as *Drosophila*, cytoophidia have been observed in stem cells and are thought to play a role in maintaining stem cell identity and function. During *Drosophila* development, the progenitor cells in the early first and second instar stages contain a huge number of cytoophidia, which gradually disappear in later stages, including the central nervous system, fat body, lymph gland, trachea, and imaginal discs [72]. Additionally, CTPS and IMPDH cytoophidia spontaneously form in undifferentiated mouse embryonic stem cells and induce pluripotent stem cells [5]. The presence and regulation of cytoophidia are linked to the metabolic demands of fast-differentiating cells.

### 4.5. Cytoskeleton-like Support

In *C. crescentus*, cytoophidia shift from the cellular center to the cell periphery and regulate the curvature of the cell body independently of their catalytic function [2]. Additionally, the CTPS^H355A^ point mutation induces follicle cell ingression and increases epithelial heterogeneity. This suggests that cytoophidia are essential for maintaining follicle epithelium integrity [84]. In many *Drosophila* tissues, cytoophidia often assemble beneath the cell cortex, including in tissues such as the testes, salivary glands, and fat body. Knockdown of CTPS leads to abnormal cell junctions, resulting in vacuole-like gaps. Additionally, both the genomic in situ point mutation CTPS^H355A^ and the overexpression of CTPS^H355A^ cause abnormal adhesion between fat body cells [85].

### 4.6. Protein Stabilization

The assembly of cytoophidia functions as a mechanism to store metabolic enzymes and increase protein stability, preventing CTPS ubiquitination and further degradation [17,86]. Notably, the CTPS protein level is critical for the formation of cytoophidia [4], and ectopic expression of CTPS induces longer and more curved cytoophidia [87]. Further, forming cytoophidia inhibits CTPS ubiquitination and further prolongs the half-life of CTPS [86]. This proved that CTPS cytoophidia functions as a metabolic stabilizer to buffer intracellular CTPS pools.

## 5. Cytoophidia in Disease

Humans have two genes, CTPS1 and CTPS2, with a similarity of 75%. Defects in the CTPS1 gene lead to reduced numbers of various types of immune cells, and the proliferation of T cells and B cells is impaired upon antigen receptor-mediated activation, resulting in severe bacterial and viral infections in patients [88,89,90]. Additionally, CTPS1 is a novel synergistic target in multiple myeloma [91] and other cancers [92]. Abnormal regulation of CTPS2 is often associated with osteosarcoma, a type of malignant bone cancer in adolescents [93]. Additionally, CTPS is an effective target for the development of antiviral, anticancer, antiprotozoal, and immunosuppressive drugs.

Thiophenecarboxamide derivatives can inhibit bacterial CTPS, thereby killing *M. tuberculosis* [94,95]. The natural product acivicin inhibits the glutamine amidotransferase activity of CTPS and other enzymes [96]. The cytidine derivative cyclopentenyl cytosine is also a CTPS inhibitor that can inhibit the proliferation of vascular smooth muscle cells (VSMCs) and the formation of neointima induced by injury, thereby inducing VSMC redifferentiation [97]. The antitumor cytidine analog gemcitabine-5′-triphosphate is a strong competitive inhibitor of CTP [98]. Additionally, CTPS is also a potential antiparasitic drug target against trypanosomiasis and toxoplasmosis [99,100].

In mammalian systems, cytoophidia are present in various cancers, including colon cancer, ovarian cancer, melanoma, prostate, and lymphoma [73]. The proliferation of tumor cells requires the synthesis of large amounts of nucleic acids, phospholipids, and proteins, leading to an increased expression or activity of many metabolic enzymes, including CTPS. Notably, hepatocellular carcinoma (HCC) samples exhibit many cytoophidia, whereas no cytoophidia were detected in adjacent non-cancerous hepatocytes [73]. Poorly differentiated HCC cells (grade 3) or slightly differentiated (grade 2) cells are more likely to exhibit CTPS cytoophidia [73]. This suggests that the CTPS cytoophidium is a metabolic adaption to the cellular stress of human hepatocellular carcinoma.

Moreover, patients with clear cell renal cell carcinoma who exhibit high IMPDH1 protein levels have shorter overall survival and disease-free survival, in which IMPDH1-assembled cytoophidia are positively associated with tumor metastasis [101]. Mechanistically, IMPDH1 cytoophidia stabilize and translocate Y-box binding protein 1 into the nucleus to promote tumor metastasis [101]. IMPDH cytoophidia can also be used to determine tumor malignancy, distinguishing between invasive acral lentiginous melanoma and nevi [102]. Therefore, modulating cytoophidia assembly to regulate enzyme activity might be a novel approach for cancer therapy.

## 6. Conclusions and Perspectives

Cytoophidia have been observed in various organisms, from bacteria to humans, and are involved in the regulation of nucleotide synthesis. The subcellular localization of proteins can effectively regulate their function, and the formation of cytoophidia by metabolic enzymes is a new form of intracellular compartmentation. Cytoophidia have been found in many core pathways including glycolysis, fatty acid synthesis, amino acid synthesis, and nucleotide synthesis [22], which are mostly responsible for the rate-limiting step. Cytoophidia in different tissues or at different subcellular locations may have distinct biological functions. They might function in maintaining the structure of normal cells, facilitate the transport of substrates and products, or be involved in other aspects of cellular physiological regulation.

These rapidly growing cells and precursor cells always have higher CTPS signal intensity and greater cytoophidia formation, which may be due to the presence of cytokines that positively regulate the assembly of cytoophidia in these rapidly proliferating cells or their high requirements for metabolic fluxes, such as nucleotides and phospholipids. Cellular metabolism is intricately calibrated, finely balanced, and robustly adaptable to signals in developmental and metabolic cues.

CTPS filaments dynamically switch between active and inactive forms in response to changes in substrate and product levels [25], which suggests filaments function as a mechanism of allosteric regulation, and play a role in regulating metabolic flux. This raises a new question: how do these two enzymatic activity states switch swiftly, and do we need electron microscopy technology to explore it? Further, whether the assembly of cytoophidia can affect the stem cell activity, proliferation, or differentiation of cells remains to be investigated.

Interestingly, cytoophidia have garnered significant attention due to their intricate structural composition and dynamic intracellular behavior. While CTPS is the primary component, cytoophidia are not solely comprised of CTPS. Studies suggest that other metabolic enzymes can also localize to these structures, potentially contributing to their function and regulation. The reticular structure of CTPS cytoophidia may provide space for other components, such as IMPDH [63], which suggests that cytoophidia may incorporate other enzymes involved in nucleotide metabolism.

Further, proteins that regulate the assembly and disassembly of CTPS filaments or those involved in post-translational modifications (e.g., ubiquitination, methylation, phosphorylation) of CTPS might also be part of the cytoophidia structure. These proteins can influence the stability and function of cytoophidia. In addition, there is emerging evidence that they can exhibit some degree of motility within the cell. Cytoophidia can be transported from nurse cells to the oocyte via ring canals [68], but this transport mechanism remains elusive.

The activity of CTPS increases in many human cancers, and cytoophidia assembly can participate in the regulation of enzyme activity, suggesting that altering cytoophidia assembly may serve as a potential drug target to avoid side effects such as neurotoxicity caused by potent inhibitors of CTPS. Currently, the electron microscopy structures of human CTPS and cytoophidia have been revealed [25,26], which greatly facilitates the development of small molecule agents for modulating the cytoophidium assembly. Research in model organisms has provided valuable insights into their roles, and ongoing studies aim to elucidate their regulation and implications for human health and disease.

## Figures and Tables

**Table 1:** List of metabolic enzymes that form filamentous structures.

Enzyme Name	Species	Discovery Year	Reference/Publication
CTP synthase (CTPS)	*Drosophila* *C. crescentus* *S. cerevisiae*	2010	Liu, J Genet Genomics, 2010 [1] Ingerson-Mahar et al., Nat Cell Biol, 2010 [2] Noree et al., J Cell Biol, 2010 [3]
Inosine monophosphate dehydrogenase (IMPDH)	*Homo sapiens*	2006	Ji et al., J Biol Chem, 2006 [40]
PRPP synthase (PRPS)	*E. coli* *Homo sapiens*	2022 2023	Hu et al., Elife. 2022 [41]; Lu et al., Cell Biosci. 2023 [42]
Glycogen debranching enzyme (GDE), thioredoxin peroxidase (TPx), asparagine synthetase (ASNS)	*S. cerevisiae*	2016	Shen et al., J Genet Genomics, 2016 [39]
Kynureninase, PRPP synthetase, GDP-mannose pyrophosphorylase	*S. cerevisiae*	2019	Noree et al., Mol Biol Cell, 2019 [43]
Delta-1-pyrroline-5-carboxylate synthase (P5CS)	*Drosophila*	2020	Zhang et al., J Genet Genomics, 2020 [32]
Glucokinase (GLK)	Yeast	2020	Stoddard et al., Science, 2020 [44]
Glutamine synthetase (GLN)	Yeast	2009	Narayanaswamy et al., Proc Natl Acad Sci, 2009 [45]
Glutamic dehydrogenase (GDH)	Bovine	1972	Josephs & Borisy, J Mol Biol, 1972 [46]
Glutaminase	Pig	1970	Olsen et al., J Mol Biol, 1970 [47]
Acetyl coenzyme A carboxylase (ACC)	Several animals	1969	Kleinschmidt et al., Science, 1969 [36]
Phosphofructokinase (PFK)	Rabbit	1971	Kemp, J Biol Chem, 1971 [48]

**Table 2:** Key regulators influencing the assembly of CTPS cytoophidia.

Regulators	Functions	Reference/Publication
Nucleotides and analogs	Directly binding.	/
mTORC1/S6K1	mTOR pathway controls CTPS cytoophidium assembly.	Sun and Liu, J Genet Genomics, 2019 [49]; Andreadis et al., J Biol Chem, 2019 [50]
AKT1	Inactivation of the AKT1 pathway induces cytoophidia formation.	Aughey et al., Biol Open, 2014 [51]
GCN2/ATF4/MTHFD2	Starvation stress and glutamine deficiency activate the GCN2/ATF4/MTHFD2 axis, thus coordinating CTPS filament formation.	Lin et al., Cell Rep, 2018 [52]
Myc	CTPsyn acts downstream of Myc.	Aughey et al., PLoS Genet, 2016 [53]
Ras	Overexpressing active Ras induces elongate and abundant cytoophidia.	Zhou et al., Exp Cell Res, 2022 [54]
Hippo	Inactivation of the Hippo pathway correlates with reduced cytoophidium.	Weng et al., Int J Mol Sci, 2024 [55]
Ack kinase	DAck localizes to CTPS filaments.	Strochlic at al., EMBO Rep, 2014 [56]
Cbl	Cbl is required for CTPsyn filament formation.	Wang et al., Genetics, 2015 [57]
Ubiquitination regulators	Ubiquitination and deubiquitination affect CTPS filamentation.	Andreadis et al., Exp Cell Res, 2022 [58]
Histone chaperone Slm9	Slm9 is required for cytoophidium biogenesis.	Feng et al., Exp Cell Res, 2022 [59]
Myo52	Myo52 is required for the active transport of cytoophidia.	Li et al., FASEB J, 2018 [60]
Polarity regulators	Knockdown of apical polarity regulators leads to cytoophidia instability and abnormal distribution.	Wang et al., Exp Cell Res, 2021 [61]
